# Telomere Shortening and Increased Oxidative Stress in Lumbar Disc Degeneration

**DOI:** 10.3390/ijms231710125

**Published:** 2022-09-04

**Authors:** Manassanan Jitjumnong, Pornpan Chalermkitpanit, Tanyawan Suantawee, Sinsuda Dechsupa, Ladawan Vajarintarangoon, Sittisak Honsawek

**Affiliations:** 1 Center of Excellence in Osteoarthritis and Musculoskeleton, Department of Biochemistry, Faculty of Medicine, Chulalongkorn University, King Chulalongkorn Memorial Hospital, Thai Red Cross Society, Bangkok 10330, Thailand; 2 Department of Anesthesiology, Faculty of Medicine, Chulalongkorn University, King Chulalongkorn Memorial Hospital, Thai Red Cross Society, Bangkok 10330, Thailand; 3 Department of Nutrition and Dietetics, Faculty of Allied Health Sciences, Faculty of Medicine, Chulalongkorn University, King Chulalongkorn Memorial Hospital, Thai Red Cross Society, Bangkok 10330, Thailand

**Keywords:** antioxidant, lumbar disc degeneration, oxidative stress, relative telomere length, severity

## Abstract

Lumbar disc degeneration (LDD) contributes to low back pain. This study aimed to determine relative telomere length (RTL), oxidative stress status, and antioxidant levels and examine the relationships between RTL, oxidative stress, and the severity in LDD patients. A total of 100 subjects, 50 LDD patients and 50 healthy controls, were enrolled in the case–control study. Blood leukocyte RTL was analyzed using quantitative real-time polymerase chain reaction. Lipid peroxidation was determined by malondialdehyde (MDA) assay. Plasma 8-hydroxy 2′-deoxyguanosine (8-OHdG) values were determined using enzyme-linked immunosorbent assay. Total antioxidant capacity (TAC) and ferric reducing antioxidant power (FRAP) in plasma were also measured. The LDD patients had significantly shorter telomeres than the healthy controls (*p* = 0.04). Blood leukocyte RTL was inversely correlated with the LDD severity (*r* = −0.41, *p* = 0.005). Additionally, plasma MDA and 8-OHdG levels were markedly greater in LDD patients than in the controls (*p* = 0.01 and *p* = 0.002, respectively). Furthermore, the plasma MDA level showed a positive correlation with the radiographic severity (*r* = 0.49, *p* = 0.001). There was a positive correlation between plasma 8-OHdG and the severity (*r* = 0.60, *p* < 0.001). Moreover, plasma TAC and FRAP levels were significantly lower in LDD patients than in the controls (*p* = 0.04). No significant differences in plasma TAC and FRAP were observed among the three groups of LDD severity. We found that RTL was negatively correlated with the severity while plasma MDA and 8-OHdG levels were positively correlated with the severity. These findings suggest that blood leukocyte RTL, plasma MDA, and 8-OHdG may have potential as noninvasive biomarkers for the assessment of severity in LDD.

## 1. Introduction

Low back pain is a prevalent musculoskeletal disorder that affects a large percentage of the population worldwide and is increasingly frequent in the elderly population. One of the underlying factors of low back pain is lumbar disc degeneration (LDD), which is characterized by the progressive wear and tear of the lumbar intervertebral disc and an inflamed disc environment. Lumbar disc degeneration may result in disc bulging, osteophytes, end-plate sclerosis, disc space narrowing, and compression and irritation of the adjacent nerve root. It may present as disc herniation, lumbar spinal stenosis, spondylolisthesis, and facet arthropathy. Clinical presentations commonly associated with lumbar disc degeneration are discogenic pain, radical pain, and weakness or numbness of the lower extremities, leading to low physical activity and poor quality of life [1]. The development of preventive or therapeutic strategies for LDD apparently relies on identification of the etiological mechanisms and biochemical markers that underlie progressive disc degeneration. Although the pathophysiology of LDD remains indeterminate, a number of risk factors that influence LDD development include aging, obesity, smoking, overuse, previous injury, and genetics [2]. In addition, several biochemical factors have been known to play crucial roles in the development of LDD.

Telomeres are the repetitive DNA repeat sequences of 5′-TTAGGG-3′, which are located at the ends of chromosomes in mammals. Telomeres are associated with shelterin, a six-protein complex, to protect the ends of chromosomes from DNA breaks and DNA degradation [3]. However, telomere shortening occurs in each DNA replication. This is due to DNA polymerase’s inability to replicate the region where the primers are placed on the DNA template, leading to cellular senescence or apoptosis [4]. A previous study has demonstrated that chronic inflammation can exacerbate telomere dysfunction by increasing reactive oxygen species (ROS)-mediated DNA damage and thus accelerate the accumulation of senescent cells in mice [5]. Additionally, senescent nucleus pulposus (NP) chondrocytes increased with age and advancing disc degeneration, whereas the telomere length and telomerase activity declined [6]. A recent study has also shown that oxidative stress inhibits proliferation and provokes premature cellular senescence in human NP cells contributing to disc degeneration [7].

Oxidative stress is an imbalance condition between oxidants and antioxidants, resulting in oxidative damage [8]. Excessive oxidants are the main cause of oxidative stress in ROS, such as superoxide anions (O_2_^−^) and hydroxyl radicals (OH^•^). These oxygen radicals cause reversible and irreversible deleterious effects on lipids, proteins, carbohydrates, and nucleic acids [9]. Oxidative DNA damage has been produced mainly by hydroxyl radicals, which are formed as 8-hydroxy-2′-deoxyguanosine (8-OHdG). Oxidative stress not only facilitates matrix degradation and inflammation but also contributes to the decrease in the number of viable and functional disc cells. DNA damage, telomere uncapping, oxidative stress, and proinflammatory cytokines can result in disc cell senescence. Moreover, senescent disc cells secrete matrix proteases, cytokines, and chemokines to deteriorate the microenvironment of discs, leading to disc degeneration [10]. Several previous studies have reported that oxidative stress plays a role in the development of lumbar degenerative disease [11,12,13]. However, little information exists regarding the associations of relative telomere length (RTL), oxidative stress, antioxidant parameters, and LDD severity.

When the balance between oxidants and antioxidants is disrupted, oxidative stress occurs. Antioxidants play an important role in protecting from oxidative damage by scavenging free radicals from the cells. Antioxidants include enzymatic antioxidants such as catalase (CAT), superoxide dismutase (SOD), and glutathione peroxidase (GPx), and nonenzymatic antioxidants such as vitamin E and vitamin C, which protect the cell against oxidative damage [14]. A previous study revealed that glutathione (GSH), an antioxidant, protected the cells against oxidative stress, which induces disc degeneration [15].

In the present study, we postulated that RTL and antioxidant status would decrease, whereas oxidative stress would increase in LDD. Therefore, the objectives of this study were to investigate the RTL, oxidative stress, and antioxidant parameters in the plasma of patients with LDD compared with the healthy controls and to examine correlations between RTL, oxidative stress, antioxidant parameters, and disease severity in LDD patients.

## 2. Results

### 2.1. Baseline Characteristics of Subjects

A total of 100 participants were recruited into the current study; there were 50 LDD patients and 50 healthy controls. In the LDD group, there were 34 females (68%) and 16 males (32%) with a mean age of 58.08 ± 2.11 years. As for the healthy controls, there were 31 females (62%) and 19 males (38%) with a mean age of 59.94 ± 0.87 years. Table 1 demonstrates the comparison of the baseline characteristics between the LDD patients and the unaffected controls. There were no significant differences regarding age, sex, and body mass index (BMI) between LDD patients and healthy controls (*p* > 0.05).

### 2.2. Shorter Relative Telomere Length in Blood Leukocytes in LDD

**Figure 1:**
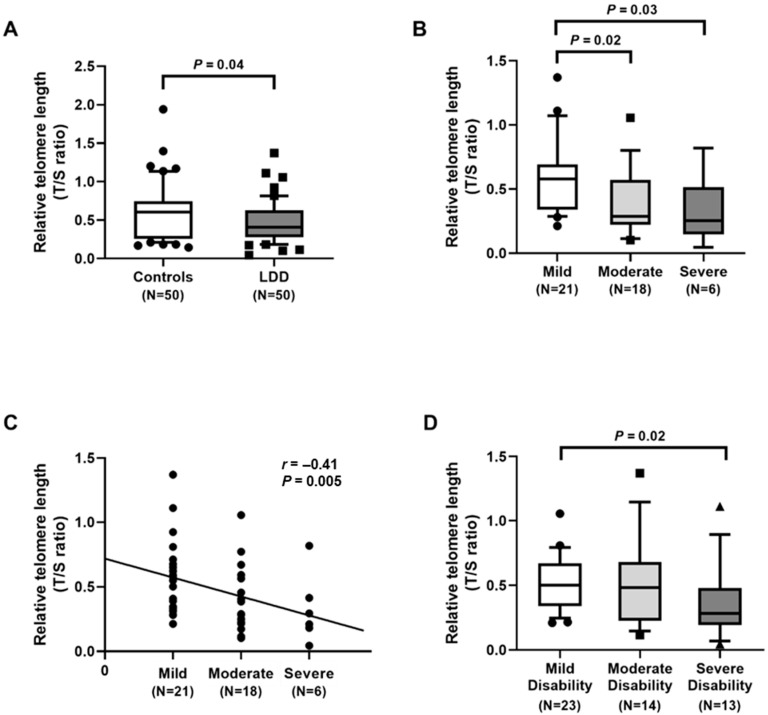
Determination of relative telomere length (RTL): (**A**) comparison of RTL in lumbar disc degeneration (LDD) patients and controls. Circles represent control group, squares LDD group; (**B**) comparison of RTL in LDD, based on the radiographic severity. Circles represent mild group, squares moderate group; (**C**) correlation of RTL and the radiographic severity. Circles represent individual data points; (**D**) comparison of RTL in LDD, stratified by Oswestry disability index score. Circles represent mild group, squares moderate group, triangles severe group.

We analyzed RTL in the blood leukocytes of LDD patients and age-matched controls. The median RTL in LDD patients was 0.41 (inter quartile range, IQR: 0.28–0.63), and the median RTL in healthy individuals was 0.61 (0.26–0.74) (Figure 1A). The blood leukocyte RTL was significantly lower in LDD subjects than in unaffected controls (*p* = 0.04), as displayed in Figure 1A. Subsequently, the LDD patients were categorized into three groups: mild, moderate, and severe groups based on the radiographic severity [16]. The RTLs in the blood leukocytes of patients in the mild (n = 21), moderate (n = 18), and severe groups (n = 6) were 0.58 (0.34–0.69), 0.29 (0.22–0.57), and 0.25 (0.15–0.52), respectively (Figure 1B). The blood leukocyte RTLs in the moderate and severe groups were significantly lower than those in the mild group (*p* = 0.02 and *p* = 0.03, respectively). Additionally, the relationship between blood leukocyte RTL and the LDD severity was statistically evaluated. The RTL in blood leukocytes was inversely correlated with the LDD severity (*r* = −0.41, *p* = 0.005) (Figure 1C). In analyses stratified by Oswestry disability index (ODI) score, patients were divided into three groups: mild disability (0–40%), moderate disability (41–60%), and severe disability (61–100%). Twenty-three patients were classified as mild disability, 14 as moderate disability, and 13 as severe disability. The results showed that LDD patients with severe disability had significantly lower RTL in blood leukocytes (*p* = 0.02) (Figure 1D).

### 2.3. Lipid Peroxidation: Malondialdehyde in Plasma

**Figure 2:**
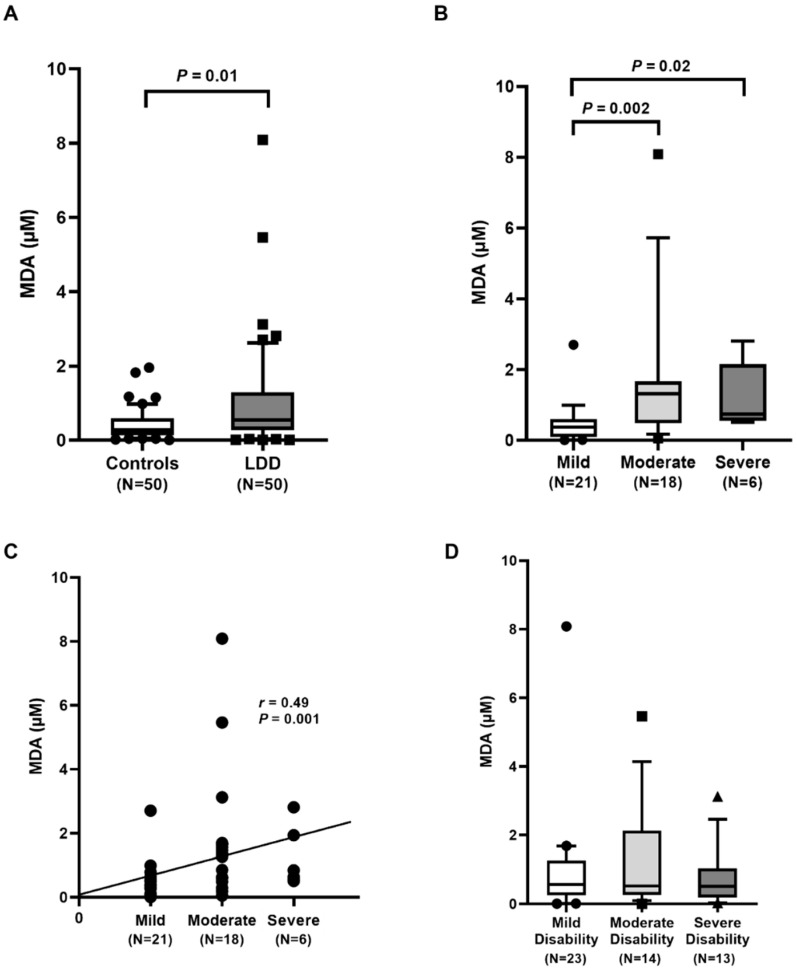
Determination of malondialdehyde (MDA) concentration: (**A**) comparison of MDA concentration in lumbar disc degeneration (LDD) patients and controls. Circles represent control group, squares LDD group; (**B**) comparison of MDA concentration in LDD, based on the radiographic severity. Circles represent mild group, squares moderate group; (**C**) correlation of MDA concentration and the severity. Circles represent individual data points; (**D**) comparison of MDA concentration in LDD, stratified by Oswestry disability index score. Circles represent mild group, squares moderate group, triangles severe group.

Increased malondialdehyde levels (MDA), an end-product of lipid peroxidation, play an important role in various degenerative diseases including LDD. MDA is considered as a good biomarker of oxidative stress. As displayed in Figure 2A, plasma MDA levels were significantly higher in LDD patients than in healthy controls (*p* = 0.01). With regard to the radiographic severity, plasma MDA levels in mild, moderate, and severe groups were 0.38 (0.10–0.60) µM, 1.32 (0.49–1.67) µM, and 0.74 (0.55–2.15) µM, respectively (Figure 2B). Plasma MDA concentration was remarkably lower in the mild group when compared to the moderate group (*p* = 0.002) and severe group (*p* = 0.02). There was no difference in plasma MDA between the moderate and severe groups. Furthermore, plasma MDA concentration showed a positive correlation with the radiographic severity (*r* = 0.49, *p* = 0.001) (Figure 2C). When classified by the ODI score, plasma MDA concentrations in patients with mild, moderate, and severe disability were 0.57 (0.26–1.27) µM, 0.53 (0.27–2.13) µM, and 0.51 (0.19–1.04) µM, respectively. No significant differences in plasma MDA between three groups were observed (Figure 2D).

### 2.4. Increased 8-Hydroxy-2′-Deoxyguanosine Measurement in Plasma

**Figure 3:**
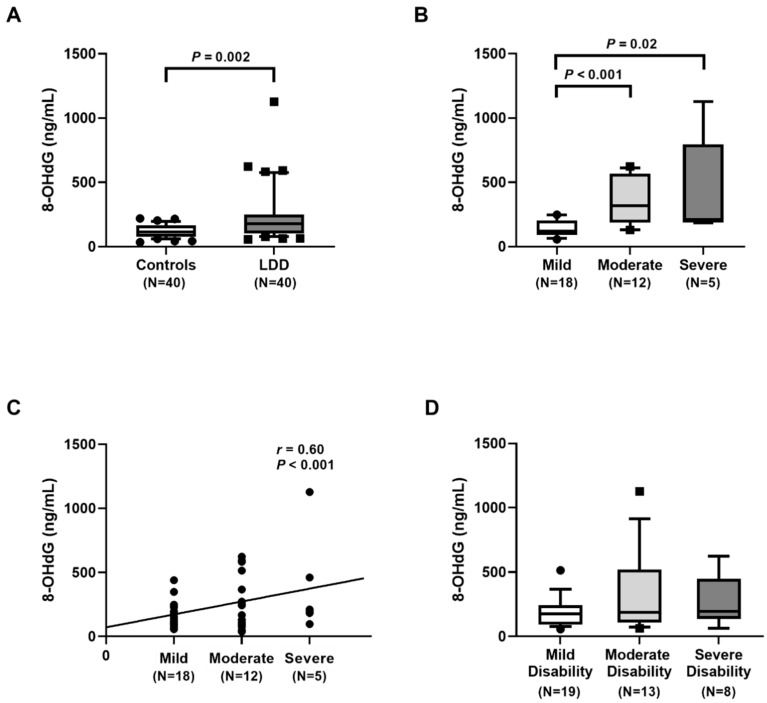
Determination of 8-hydroxy-2′-deoxyguanosine (8-OHdG) concentration: (**A**) comparison of 8-OHdG concentration in lumbar disc degeneration (LDD) patients and controls. Circles represent control group, squares LDD group; (**B**) comparison of 8-OHdG concentration in LDD, based on the disease severity. Circles represent mild group, squares moderate group; (**C**) correlation of 8-OHdG concentration and the radiographic severity. Circles represent individual data points; (**D**) comparison of 8-OHdG concentration in LDD, stratified by Oswestry disability index score. Circles represent mild group, squares moderate group.

We further determined 8-hydroxy-2′-deoxyguanosine (8-OHdG) status being a biomarker of oxidative DNA damage in the plasma of LDD subjects (n = 40) and healthy controls (n = 40). The results showed that the median plasma 8-OHdG level in LDD patients was also markedly greater than in the control participants (179.28 (101.88–247.55) ng/L vs. 115.76 (77.12–164.79) ng/L, *p* = 0.002) (Figure 3A). Plasma 8-OHdG levels in the moderate and severe groups were substantially higher than those in the mild group (*p* < 0.001 and *p* = 0.02, respectively) (Figure 3B). Subsequent analysis revealed that there was a positive correlation between 8-OHdG concentration and the severity (*r* = 0.60, *p* < 0.001), as demonstrated in Figure 3C. There was no significant difference in plasma 8-OHdG between the three groups regarding the ODI severity (Figure 3D).

### 2.5. Total Antioxidant Capacity in Plasma

**Figure 4:**
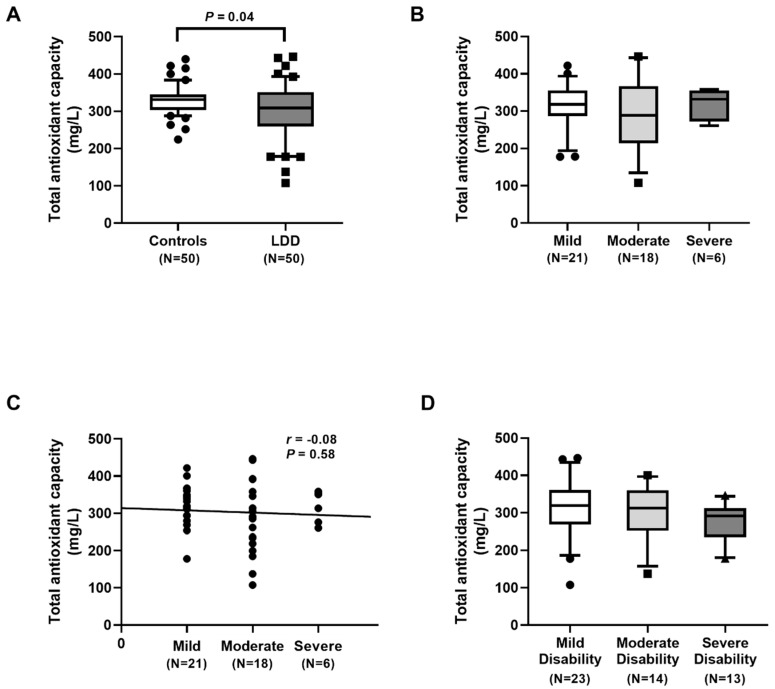
Determination of total antioxidant capacity (TAC): (**A**) comparison of TAC in lumbar disc degeneration (LDD) patients and controls. Circles represent control group, squares LDD group; (**B**) comparison of TAC in LDD, based on the radiographic severity. Circles represent mild group, squares moderate group; (**C**) correlation of TAC and the radiographic severity. Circles represent individual data points; (**D**) comparison of TAC in LDD, stratified by Oswestry disability index score. Circles represent mild group, squares moderate group, triangles severe group.

Low antioxidant levels contribute to more oxidative damage to cells. As shown in Figure 4A, plasma TAC levels in LDD patients and controls were 308.44 (258.91–351.48) mg/L and 331.27 (303.14–344.95) mg/L, respectively. Plasma TAC in LDD subjects was significantly lower than that in controls (*p* = 0.04). There was no significant difference between the three groups of severity, and no correlation between TAC and the severity was observed (Figure 4B–D).

### 2.6. Ferric reducing Antioxidant Power in Plasma

**Figure 5:**
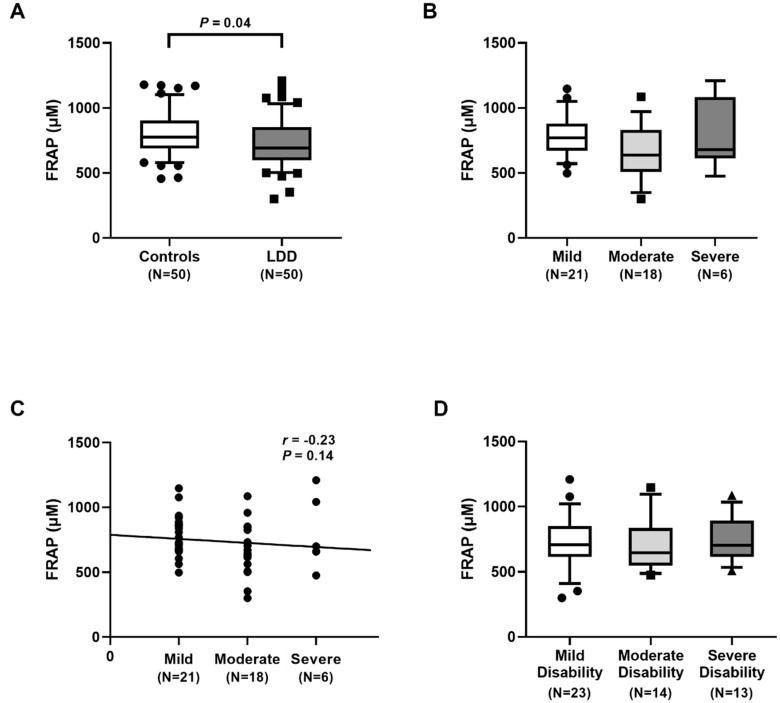
Determination of ferric reducing antioxidant power (FRAP) values: (**A**) comparison of FRAP values in lumbar disc degeneration (LDD) patients and controls. Circles represent control group, squares LDD group; (**B**) comparison of FRAP values in LDD, based on the radiographic severity. Circles represent mild group, squares moderate group; (**C**) correlation of FRAP values and the radiographic severity. Circles represent individual data points; (**D**) comparison of FRAP values in LDD, stratified by Oswestry disability index score. Circles represent mild group, squares moderate group, triangles severe group.

As displayed in Figure 5A, plasma FRAP values were significantly lower in LDD patients than in controls (690.00 (597.50–852.64) µM vs. 775.07 (689.50–905.86) µM, *p* = 0.04). According to the severity of radiography and disability, plasma FRAP levels were not significantly different between groups. No association between plasma FRAP and the severity was detected, as demonstrated in Figure 5B–D.

## 3. Discussion

LDD is the most common cause of low back pain caused by degenerative and oxidative processes. The degeneration of the lumbar spine results from the over-compression of the lumbar disc due to aging, traumatic injury, overweight, and poor body mechanics [2]. The pathophysiology of LDD remains elusive; these may include biomechanical, biochemical, and genetic factors. Therefore, the present study was designed to examine blood leukocyte RTL, oxidative stress, and antioxidant levels in LDD.

Telomeres have been known to play the potential role in protection and maintain telomere integrity. With age and degeneration, telomeres in human somatic cells normally decline with each round of cell proliferation. Since the telomeres progressively shorten with increasing age, the RTL has attracted attention recently for use as a biomarker for age-related diseases [17,18]. However, little attention has been paid to exploit the relationships of RTL, oxidative stress, and antioxidants in LDD. In the current study, we revealed that blood leukocyte RTL in LDD patients was significantly lower than that in controls. The reduction of RTL was observed in more severity of disease. Furthermore, the negative correlation between RTL and the LDD severity was noted. This finding is consistent with a previous study, where Le Maitre et al. reported that the mean telomere length was significantly decreased in patients with degenerative discs when compared with non-degenerative discs [19]. Mean telomere length declined significantly with advancing age in cells from non-degenerative discs and also reduced with progressive stages of degeneration [19]. Moreover, Dechsupa et al. highlighted that hypertrophic ligamentum flavum (LF) tissue from lumbar stenosis patients presented shorter RTL than non-hypertrophic LF tissue [20]. The results of this study suggest that RTL in blood leukocytes could be applied as a noninvasive biochemical marker reflecting the severity of LDD.

Degenerative diseases and aging have been linked to an increase in ROS production and radicals in the cells. The markers of oxidative stress that were determined in the present study include MDA and 8-OHdG. The increased amounts of free radicals can result in excessive lipid peroxidation, indicated by a rise in plasma MDA. Our results showed that circulating MDA in LDD patients was significantly higher than that in the controls. The elevation of plasma MDA was found with increasing severity of LDD. Moreover, the positive correlation of plasma MDA and the disease severity was evidenced. In accordance with other findings, circulating MDA concentration was significantly higher in LDD patients than in the controls [12]. In addition to oxidative damage, MDA level in the degenerative intervertebral disc of rats was also significantly higher when compared to that in the controls [21]. Thus, LDD patients with low RTL and high oxidative stress may be exposed to a higher risk of more severe disease.

In this study, we found significantly higher plasma 8-OHdG levels in LDD patients than in controls. Moreover, plasma 8-OHdG was positively correlated with the severity. These findings are in agreement with previous report, showing that the circulating 8-OHdG level was significantly higher in LDD patients than in the control group [22]. Additionally, there was a positive correlation between the circulating 8-OHdG and ODI parameter. Furthermore, 8-OHdG value in hypertrophic LF tissues was also significantly higher than that in non-hypertrophic tissues [20]. The precise underlying mechanisms whereby blood leukocyte RTL in LDD patients is shorter than that in healthy controls remain uncertain. It could be attributed to chronic inflammation and oxidative stress leading to telomeric DNA damage to the cells, which then promotes the shortening of RTL [23]. Telomeric DNA sequences, rich in guanine residues, are plausibly more susceptible to oxidative stress, especially by the formation of 8-OHdG. Furthermore, these could promote DNA double-strand breaks specifically at telomeric regions leading to the loss of the distal fragments of telomeric DNA and telomere shortening [24]. Additionally, oxidative stress along with proinflammatory cytokines could trigger the activation of matrix metalloproteinases, which can directly degrade the extracellular matrix of the disc rendering its degeneration. The mechanisms underlying the connection of telomere attrition, oxidative stress, and proinflammatory cytokines both in systemic and local tissues require further intensive research.

Our results showed an excessive degree of oxidative stress in LDD subjects, as was evidenced by an increment in oxidative stress parameters and a reduction in antioxidant parameters. Additionally, plasma TAC levels were significantly lower in LDD patients than in the controls. Moreover, plasma FRAP levels were also significantly decreased in the LDD patients. Plasma TAC and FRAP, known as antioxidant parameters, were utilized to measure the total antioxidant status. In accord with this study, Suantawee and colleagues reported that plasma Trolox equivalent antioxidant capacity and FRAP levels in patients with degenerative joint disease were significantly lower than those in the healthy controls [25]. Furthermore, Bakirezer et al. unveiled that circulating glutathione reductase, one of the antioxidant biomarkers, in LDD patients was lesser than that in healthy controls [13]. However, we noticed no significant difference in plasma TAC and FRAP among the three groups of severity in LDD. A possible explanation for no correlation of plasma TAC and FRAP among the LDD severity might be attributed to differences in populations, disease stages, or measurements applied or to incomplete control of confounding variables.

The present research acknowledges potential limitations that should be considered. First, this study was cross-sectional in its design, with relatively small numbers of patients and controls. Therefore, cause and effect relationships cannot be determined. However, with a small sample size, caution must be applied, as the findings might not be transferable to other populations. Multi-center prospective longitudinal studies with a large sample size are warranted to elucidate the possible relationships. Second, local intervertebral disc tissues were not collected, resulting in no direct comparison between the local tissue specimens and blood samples being made between the patients and controls. In fact, since plasma is a pool for all oxidative events in the body and it is uncertain that the plasma oxidative changes originated entirely from the inflamed disc, the results obtained from this study may not be directly extrapolated to the clinical situation. More studies on local intervertebral disc specimens are warranted for a better understanding of our findings. Third, telomere length and oxidative stress parameters may be confounded by other factors such as environment exposures, smoking, ethnicity, and lifestyle habits. Unfortunately, such information would be unavailable due to limitations of records accessibility. Forth, we mainly measured plasma oxidative stress and antioxidant status but not plasma proinflammatory cytokines. Therefore, additional research on plasma proinflammatory cytokines will be necessary to elucidate the pathogenesis of telomeric DNA damage and telomere shortening. Lastly, insufficient assessment of possible confounding factors such as medical comorbidities and body habitus need to be taken into consideration. Additional clinical and lifestyle information might be useful when interpreting the values of RTL and oxidative stress parameters.

In conclusion, LDD patients had a significantly lower RTL and higher oxidative stress, as evident by higher MDA and 8-OHdG concentrations and lower TAC and FRAP values than the healthy controls. The telomere shortening and high oxidative stress were observed in more severity of LDD. Blood leukocyte RTL was negatively correlated with the disease severity while plasma MDA and 8-OHdG levels were positively associated with the LDD severity. These findings suggest that blood leukocyte RTL, plasma MDA, and 8-OHdG may have potential as noninvasive biomarkers for the assessment of the severity in lumbar degenerative disc disease.

## 4. Materials and Methods

### 4.1. Participants

This case–control prospective study was carried out between July 2021 and June 2022. This study included a total of 100 participants: 50 patients with LDD and 50 healthy individuals for control group. All participants were recruited from the Pain Alleviation Clinic of our hospital. None of the healthy individuals had ever experienced symptoms of back pain or radiculopathy. Concerning the clinical assessment of patients, neurological examination, lumbar magnetic resonance imaging (MRI), visual analog scale (VAS) scoring to define the pain level, and Oswestry disability index (ODI) scoring were carried out. The demographic characteristics of the cases and controls were extracted from their medical records. The inclusion criteria of the patient group were lower back pain and radiculopathy related to either LDD or lumbar disc herniation diagnosed with lumbar MRI. Participants were excluded if they had a history of previous lumbar surgery, epidural steroid injection within the last 6 months, or a history of trauma, recent infections, congenital anomalies, osteoporosis, cancers, vertebral fractures, or spinal deformities, or were pregnant or breast feeding in the time of recruitment. Subjects were also excluded if they suffered from atherosclerosis, hypertension, diabetes mellitus, cigarette smoking, alcohol abuse, and/or other chronic diseases.

This study was conducted following the guidelines of the Declaration of Helsinki and approved by the Institutional Review Board on Human Research of the Faculty of Medicine, Chulalongkorn University (1007/64). Written informed consent was acquired from each participant prior to entering into the study.

### 4.2. Radiographic Severity Assessment

MRI scans of T2-weighted spin-echo images at the lumbosacral spine were examined for degeneration severity. Severity was classified using Pfirrmann grade (I–V) [26]. Grade I represents a healthy disc possessing a bright hyperintense white signal intensity with clear separation of the NP and annulus fibrosus (AF) and normal disc height. Grade V represents a hypointense black signal intensity with no clear distinction between the NP and AF and a collapsed disc space. In the present study, LDD patients with Pfirrmann grade ≥ II were recruited. Pfirrmann grade II indicates mild change group, while Pfirrmann grade III indicates moderate change group, with Pfirrmann grade IV and V implicating severe change group.

### 4.3. Determination of Oswestry Disability Index

Oswestry disability index (ODI) is self-administered questionnaire mostly used in the assessment of low back pain. Disability due to back pain was assessed by Oswestry disability index (ODI 0–100), which ranges from 0, no disability, to 100, maximal possible disability [27]. A higher score reflects more disability. In this study, the ODI was divided into 3 groups, which are mild disability (0–40%), moderate disability (41–60%), and severe disability (61–100%).

### 4.4. Plasma and DNA Preparation

Following overnight fasting, venous blood samples were collected in Vacutainer tubes from all participants, which were immediately centrifuged and stored at −80 °C until further measurements. DNA was extracted from the buffy layer using the GF-1 nucleic acids extraction kit (Vivantis, Buckinghamshire, Malaysia). The concentration of DNA was measured using Nanodrop 2000 spectrophotometer (Thermo Scientific, Wilmington, DE, USA). Sample storage was at −80 °C until later analyses.

### 4.5. Measurement of Relative Telomere Length

Relative telomere length was performed by quantitative real-time polymerase chain reaction (real-time qPCR) [28]. The DNA was used at a final concentration of 1.5 ng/µL. The single-copy gene refers to the 36B4 gene, which encodes the acid ribosomal phosphoprotein. The ratio (T/S) is proportional to the average telomere length. PCRs were performed using StepOnePlus™ Real-Time PCR System (Applied Biosystems, Foster City, CA, USA) with SYBR Green fluorescence (RBC Bioscience, Taipei, Taiwan). Briefly, two pairs of primers were used to amplify the telomere repeats copy number relative to another 36B4 for the amplification of the single-copy nuclear gene. Primers used were as follows: telomere (accession number 01051619, amplicon size 84 bp) (forward) 5′-CGGTTTGTTTGGGTTTGGGTTTGGGTTTG GGTTTGGGTT-3′; telomere (reverse) 5′-GGCTTGCCTTACCCTTACCCTTACCCTTACCC TTACCCT-3′; 36B4 (amplicon size 75 bp) (forward) 5′-CAGCAAGTGGGAAGGTGTAATCC-3′; and 36B4 (reverse) 5′- CCCATTCTATCATCAACGGGTACAA-3′. The thermal cycling profile was 95 °C for 10 min, followed by 40 cycles at 95 °C for 15 s and 54 °C for 1 min. Relative telomere length was evaluated according to the ratio of the telomere repeat copy number (T) to the single-copy gene copy number (S) in each sample. Relative telomere length was measured using the estimation of comparative Ct method (2^−ΔΔ*C*t^ method) using the following equation: relative telomere length = 2^−ΔΔ*C*t^ (ΔΔ*C*t = (Ct_36B4,Reference_ − Ct_telomere,Reference_) − (Ct_36B4,Unknown_ − Ct_telomere,Unknown_)).

### 4.6. Determination of Lipid Peroxidation

To determine lipid peroxidation, the thiobarbituric acid-reactive-substances (TBARS) assay was used. This assay measures MDA as a secondary product of lipid peroxidation. Briefly, 200 µL of plasma was added to clean microtube with 40 µL of 2,6-di-tert-butyl-4-methyphenol (BHT) to stop over-peroxidation. Then, 400 µL of 15% trichloroacetic acid (TCA) was applied and centrifuged at 4 °C for 3 min to precipitate the protein. Next, the supernatant was collected and reacted with 400 µL of 2-thiobarbituric acid (TBA) to produce a pink-colored product. After that, the absorbance was measured at 532 nm. Malondialdehyde tetrabutylammonium salt was used as the standard. The concentration of MDA was quantified using the equation from the MDA concentration standard curve.

### 4.7. Quantitation of 8-Hydroxy-2′-Deoxyguanosine

Plasma 8-OHdG levels were analyzed using a commercially available sandwich enzyme-linked immunosorbent assay (ELISA) kit (ab285254, Abcam, Cambridge, UK). According to the manufacturer’s instruction, 50 µL of standard and plasma were applied to wells. Before washing step, 50 µL of biotinylated detection antibody was added and incubated at 37 °C for 45 min. After that, 100 µL of horseradish peroxidase–streptavidin conjugate was pipetted into wells and incubated at 37 °C for 30 min, followed by washing step. Next, 90 µL of substrate was added and incubated at 37 °C in the dark for 15–30 min. Finally, 50 µL of stop solution was added and the absorbance was measured at 450 nm. A standard optical density–concentration curve was generated for assessment of plasma 8-OHdG value expressed in ng/mL.

### 4.8. Analysis of Total Antioxidant Capacity

Total antioxidant capacity in plasma was assessed by vitamin C equivalent antioxidant capacity (VCEAC) assay, which was developed by Miller et al. [29] and modified by Kim et al. [30], and measures the 2,2′-azino-bis(3-ethylbenzthiazoline-6-sulphonic acid) (ABTS) radical chromogen at 734 nm, using 2,2′-azobis (2-amidinopropane) dihydrochloride (AAPH) as a thermolabile water-soluble radical initiator. Briefly, 10 µL of 5 mM ABTS reagent (Sigma-Aldrich, St. Louis, MO, USA) was mixed with 10 µL of 2 mM AAPH reagent (Merck Millipore, Massachusetts, USA) and incubated at 68 °C for 40 min to transforming ABTS to ABTS radical (ABTS^•+^), resulting in blue/green reagent. Next, blue/green ABTS^•+^ solution was diluted with phosphate buffered saline (PBS) until reaching an absorbance of 0.650 ± 0.02 at 734 nm. The reaction begins with 5 µL of plasma, and serial concentrations of standard vitamin C were added to each well. Then, 295 µL of ABTS^•+^ solution was applied and incubated at 37 °C in the dark for 10 min. The color intensity was measured at 734 nm, and VCEAC was quantified using the equation from the vitamin C concentration standard curve. The reduction of ABTS radical chromogen is proportional to the TAC in plasma. The results were expressed as mg vitamin C equivalent (VCE) per liter plasma.

### 4.9. Determination of Ferric Reducing Antioxidant Power

For measurement of plasma antioxidant power, FRAP assay was used. The FRAP assay determines the ability of the sample to reduce ferric iron to ferrous iron in a low-pH environment. A colored ferrous–tripyridyltriazine complex is formed during this process and has a maximum absorbance of 595 nm. To prepare FRAP reagent, sodium acetate buffer solution, 10 mM 2,4,6-tripyridyl-S-triazine (TPTZ) (Sigma-Aldrich, St. Louis, MO, USA), and 20 mM FeCl_3_ were mixed. The reaction begins with 10 µL of plasma, and serial concentrations of standard FeSO_4_ were applied to each well. Next, 190 µL of FRAP reagent was added and incubated at room temperature for 30 min in the dark. The absorbance was measured at 595 nm, and the concentration of FRAP was quantified using the equation from the FeSO_4_ concentration standard curve. The results are expressed as μmol Trolox per liter plasma.

### 4.10. Statistical Analysis

Statistical analyses were performed using the Statistical Package for the Social Sciences (SPSS) version 22.0 (SPSS, Inc., Chicago, IL, USA), and figures were constructed using GraphPad Prism version 7.0. The data were presented as n (%), mean ± standard error of the mean (SEM) or median and interquartile range (IQR). The comparisons of data between LDD and controls were analyzed by chi-square test, unpaired Student *t*-test, and Mann–Whitney *U*-test where appropriate, and one-way analysis of variance or Kruskal–Wallis H test was employed for comparisons of continuous variables among LDD subgroups. Spearman’s rank correlation coefficient test was used to estimate relationships between RTL, plasma MDA, 8-OHdG, TAC, FRAP, and severity. *p*-values less than 0.05 were considered statistically significant for differences and correlations.

## Figures and Tables

**Table 1:** Baseline characteristics of LDD patients and healthy controls.

Characteristics	LDD	Controls	*p*-Values
	(n = 50)	(n = 50)	
Age (years)			
Mean (SEM)	58.08 (2.11)	59.94 (0.87)	0.42 ^b^
Gender			0.53 ^a^
Male, n (%)	16 (32.00)	19 (38.00)	
Female, n (%)	34 (68.00)	31 (62.00)	
BMI (kg/m^2^)			
Mean (SEM)	25.92 (0.60)	24.53 (0.60)	0.11 ^b^
Radiographic assessment			
Mild, n (%)	21 (46.67)	-	
Moderate, n (%)	18 (40.00)	-	
Severe, n (%)	6 (13.33)	-	
Visual analogue scale (VAS)			
Mean (SEM)	6.34 (0.27)	-	
Oswestry disability index (ODI)			
Mean (SEM)	47.60 (2.49)	-	
Oswestry disability index (ODI)			
Mild disability, n (%)	23 (46.00)	-	
Moderate disability, n (%)	14 (28.00)	-	
Severe disability, n (%)	13 (26.00)	-	
Duration of pain			
Acute (<1 month), n (%)	2 (4.00)	-	
Subacute (1–3 months), n (%)	8 (16.00)	-	
Chronic (>3 months), n (%)	40 (80.00)	-	

^a^ Chi-square test. ^b^ Unpaired Student *t*-test.

## Data Availability

The data presented in this study are available on request from the corresponding authors.

## Abbreviations

AAPH	2,2′-azobis (2-amidinopropane) dihydrochloride
ABTS	2,2′-azino-bis(3-ethylbenzthiazoline-6-sulphonic acid)
AF	Annulus fibrosus
BHT	2,6-di-tert-butyl-4-methyphenol
CAT	Catalase
ELISA	Enzyme-linked immunosorbent assay
FRAP	Ferric reducing antioxidant power
GPx	Glutathione peroxidase
GSH	Glutathione
H_2_O_2_	Hydrogen peroxide
IQR	Interquartile range
LDD	Lumbar disc degeneration
LF	Ligamentum flavum
MDA	Malondialdehyde
NP	Nucleus pulposus
8-OHdG	8-hydroxy 2′-deoxyguanosine
O_2_^−^	Superoxide anion
ODI	Oswestry disability index
OH^•^	Hydroxyl radical
PBS	Phosphate buffered saline
qPCR	Quantitative real-time polymerase chain reaction
ROS	Reactive oxygen species
RTL	Relative telomere length
S	Single-copy gene copy number
SEM	Standard error of the mean
SOD	Superoxide dismutase
SPSS	Statistical Package for the Social Sciences
T	Telomere repeat copy number
TAC	Total antioxidant capacity
TBA	2-thiobarbituric acid
TBARS	Thiobarbituric acid-reactive-substances assay
TCA	Trichloroacetic acid
TPTZ	2,4,6-tripyridyl-S-triazine
VAS	Visual analog scale
VCE	Vitamin C equivalent
VCEAC	Vitamin C equivalent antioxidant capacity
